# Highly Efficient Photocatalytic Hydrogen Production of Flower-like Cadmium Sulfide Decorated by Histidine

**DOI:** 10.1038/srep13593

**Published:** 2015-09-04

**Authors:** Qizhao Wang, Juhong Lian, Jiajia Li, Rongfang Wang, Haohao Huang, Bitao Su, Ziqiang Lei

**Affiliations:** 1 College of Chemistry and Chemical Engineering, Northwest Normal University, Key Laboratory of Eco-Environment-Related Polymer Materials, Ministry of Education of China, Key Laboratory of Gansu Polymer Materials, Lanzhou 730070, China; 2 College of Materials Science and Engineering, South China University of Technology, Guangzhou, 510640, China; 3 Key Laboratory of Green Catalysis of Higher Education Institutes of Sichuan, College of Chemistry and Pharmaceutical Engineering, Sichuan University of Science and Engineering, Zigong, 643000, China

## Abstract

Morphology-controlled synthesis of CdS can significantly enhance the efficiency of its photocatalytic hydrogen production. In this study, a novel three-dimensional (3D) flower-like CdS is synthesized via a facile template-free hydrothermal process using Cd(NO_3_)_2_•4H_2_O and thiourea as precursors and L-Histidine as a chelating agent. The morphology, crystal phase, and photoelectrochemical performance of the flower-like CdS and pure CdS nanocrystals are carefully investigated via various characterizations. Superior photocatalytic activity relative to that of pure CdS is observed on the flower-like CdS photocatalyst under visible light irradiation, which is nearly 13 times of pure CdS. On the basis of the results from SEM studies and our analysis, a growth mechanism of flower-like CdS is proposed by capturing the shape evolution. The imidazole ring of L-Histidine captures the Cd ions from the solution, and prevents the growth of the CdS nanoparticles. Furthermore, the photocatalytic contrast experiments illustrate that the as-synthesized flower-like CdS with L-Histidine is more stable than CdS without L-Histidine in the hydrogen generation.

Recently, morphology-controlled synthesis of semiconductor nano-/micro-crystals has attracted essential interests because it enables the development of the addition of flexibility to existing systems in many areas, such as catalysis, optics, magnetism, biology, and so on^1^. Until now, mono-morphological structures (dot^2^, wire^3^, tube^4^, etc.) have been obtained. Hierarchical structure-based morphologies, such as comb-like^5^, dendrite-like^6^, snowflake-like^7^, flower-like^8^, rod-like^9^ and urchin-like^10^ structures, show unique properties by combining the features of micrometer- and nanometer-scaled building blocks in one crystal.

Controlling the shape of nanocrystalline materials is a crucial issue in the exploitation of novel properties in nanoscience research. CdS is one of the II–VI group semiconductor materials with a direct band gap of 2.4 eV^11^, which represents an important field for the potential utilization in nonlinear optics and photocatalysis^12^. Therefore, it is significant to make considerable efforts to synthesize CdS nano-/micro-crystals of different morphologies. Biomolecule-assisted routes have been widely used in the preparation of various nanomaterials, whose special structures and fascinating self-assembling functions allow them to serve as templates for the design and preparation of complicated structures^13^. Among many biomolecules, amino acids can give rise to complex three dimensional structures through disulfide bonds or crosslinked amino acids^14^. Qian *et al.* has reported a facile L-cysteine-assisted method for synthesis of CdS in spherical nanostructures^15^. However, there are few reports on synthesis of CdS crystals by using a bio-compatible organic chelating agent L-Histidine and it differs from other amino acids by an imidazole side group. The imidazole side groups of histidine play an important role for chelation of ions^16^. Chelating agents are materials which capture metal ions from a solution and form a complex to prevent further growth or agglomeration of nanoparticles. In this paper, we report the formation of CdS nanoparticles using L-Histidine as a structure-guiding agent in the hydrothermal process. L-Histidine is found to significantly influence the morphologies of CdS crystals and can improve the efficiency of photocatalytic hydrogen production. The flower-like CdS prepared by us has a high photocatalytic activity, which is nearly 13 times of that for the pure in-house CdS, for the enhanced time. It is higher than urchin-like CdS which is 4 times of that for the hollow spheres CdS^10^.

## Results

### XRD analysis

**Figure 1:**
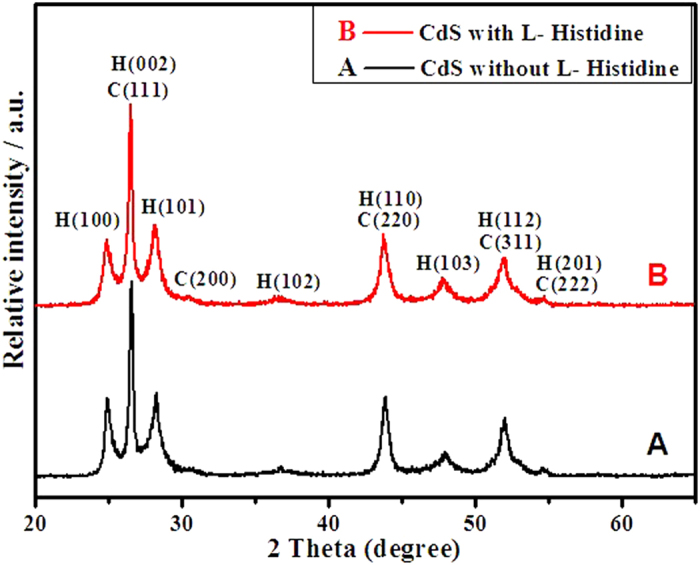
XRD patterns of CdS prepared without L-Histidine (**a**) and with L-Histidine (**b**) (H: hexagonal phases and C: cubic phases).

XRD analysis is used to investigate the phase structure and crystalline size of the products. Figure 1 presents a comparison of XRD patterns of the as-prepared CdS samples without L-Histidine and with L-Histidine. The positions of the diffraction peaks of samples match well with the theoretical values of the hexagonal structure of CdS (JCPDS No: 41–1049) with the lattice parameters α = 4.140 Å. In addition, cubic structure of CdS typically has a characteristic peak at 30.6° corresponding to the (200) plane with the cell constant α = 5.818 Å (JCPDS No: 10–0454). It clearly indicates that cubic and hexagonal phases coexist in samples.

### Morphology and formation mechanism of the flower-like CdS

**Figure 2:**
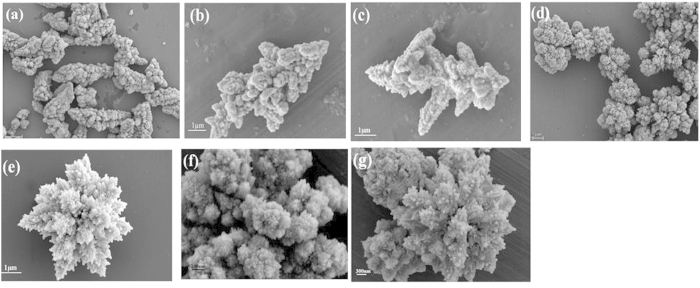
SEM images of CdS prepared without L-Histidine (**a–c**) and with L-Histidine (**d–f**), and SEM image of CdS prepared with L-Histidine after 5 h of reaction under the visible-light irradiation (**g**).

The morphology of the as-synthesized 3D CdS nanoarchitectures was examined by SEM and TEM. Figure 2(a–c) show SEM images of CdS prepared without L-Histidine, showing irregularity of dendritic architecture. Figure 2d,e show that CdS has flower-like morphology by synthesizing with the assistance of L-Histidine. Figure 2f gives out an amplifying representative flower-like morphology. It is interesting to note that the flower-like CdS nanostructures consisted of CdS nanorods, which protrude from the root of the flower-like CdS. Figure 2g proves that the morphology of CdS flower-like does not change after 5 h of reaction under the visible-light irradiation, indicating that the flower-like morphology is stable during the visible-light irradiation. In order to check the influence of L-Histidine on the morphology of CdS, different concentrations of the reagents (Cd precursor and histidine) are designed, as shown in Figure S1, the ratio of Cd precursor to histidine is 10:1, 10:2, 10:3, 10:5, and 10:10, respectively. Figure S1c presents a flower-like CdS, in which the ratio of Cd precursor to L-histidine is 10:3. The morphologies in Figure S1a and Figure S1b are similar to the one in Figure S1c, but CdS in Figure S1a and Figure S1b aggregates and has poorer crystallinity. Figure S1d and Figure S1e indicate that CdS has cauliflower-like structure when the ratio between Cd precursor and L-histidine is 10:5 or 10:10. The morphologies of products are controlled by the rates of nucleation and crystal growth. When the rate of crystalline nucleation is greater than that of crystal growth, the particle sizes will be small and the particle has low aggregation. Contrarily, rapid crystal growth will generate large particle sizes and heavy aggregation. By using the high concentration of L-histidine, it could accelerate the crystal growth to be faster than crystalline nucleation. Thus the concentration of L-histidine influences the morphology of CdS. So the optimal ratio of Cd precursor and histidine is10:3.

**Figure 3:**
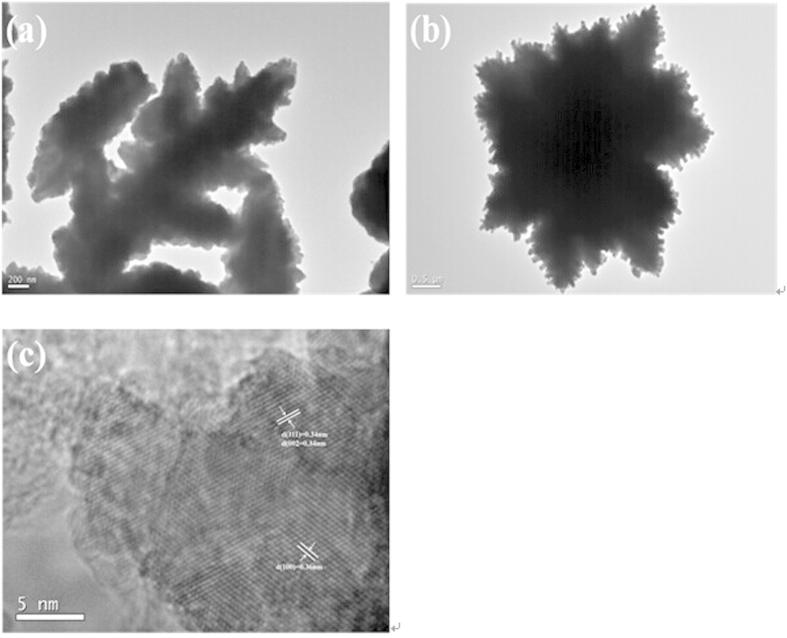
Morphological characterization of flower-like CdS. TEM images of pure CdS (**a**) and flower-like CdS (**b**); the HRTEM image of a single petal flower-like CdS (**c**).

Figure 3(a–b) shows the typical TEM images of the reference CdS and flower-like CdS. It can be found that the pure CdS has the irregularity of dendritic architecture. However, TEM image (Fig. 3b) displays that L-Histidine-assisted method for synthesis of CdS does generate flower-like structure and CdS nanorods exist in the flower-like structure. In addition, two main distinct lattice fringes are clearly distinguished in the HRTEM image (Fig. 3c). The spacing of 0.34 nm can be ascribed to the (111) and (002) crystal faces of cubic phase CdS and hexagonal phase CdS, and the spacing of 0.36 nm matches well with the (100) crystal face of hexagonal phase CdS, respectively, according to the XRD of cubic and hexagonal phases in samples.

**Figure 4:**
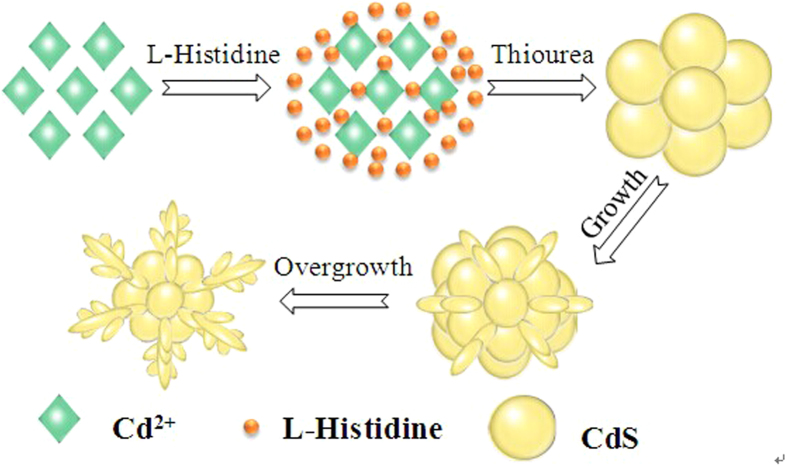
Schematic of the growth process of flower-like CdS (This Figure was drawn by our co-author Jiajia Li).

**Figure 5:**
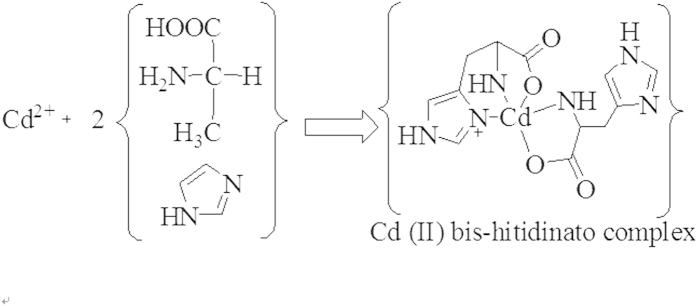
The structure of Cd(II) bis-hitidinato complex.

On the basis of the SEM observation and the experimental process, a possible mechanism is proposed to explain the formation of flower-like CdS, as depicted in Figure 4. Firstly, the imidazole rings of L-Histidine capture Cd ions to form Cd (II) bis-hitidinato complex (Figure 5) and prevent the agglomeration of CdS by releasing cadmium ions slowly into the solution^17^,^18^. And then, Cd^2+^ and thiourea might form Cd-thiourea complex because of the strong coordinating capacity of the thiourea. With increasing temperature during the hydrothermal process, the Cd-thiourea complex may tend to decompose to release active Cd^2+^ and S^2–^. Then the active Cd^2+^ reacts with active S^2–^ to form CdS seeds. L-Histidine plays a key role in the formation of flower-like CdS. The nitrogen atom of the imidazol ring promotes its solubility in aqueous solution and is also an important element to produce hydrogen-bond interaction between particles^19^. At the beginning of reaction, the number of CdS particles is small, and L-Histidine can effectively chelate most surfaces of the originally formed aggregates CdS. When the particles closely contact with each other, diffusion-controlled growth occurs in the spherical aggregates, and the surface area is reduced by particle fusion and structure rearrangement^20^. At this stage, some spheres with a rough surface are obtained. With the formation of more and more CdS nanoparticles, the petals appear from the primal multiplayer surface structure and then flower-like structures are generated. This mechanism is similar to the oriented attachment, which was observed in a system where small particles were coated with small molecules and the molecules allowed them to get close to each other and to facilitate attachment^21^.

### UV-vis absorption spectra and Nitrogen adsorption-desorption isotherms of the samples

**Figure 6:**
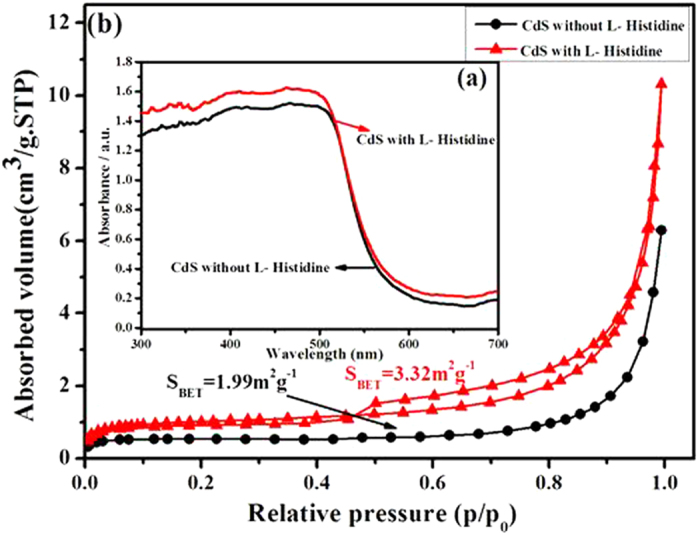
UV-vis absorption spectra (a) and Nitrogen adsorption-desorption isotherms (b) of the samples.

Figure 6a presents UV-vis absorption spectra of the samples. The absorption intensity of pure CdS without L-Histidine starts to increase rapidly at ca. 550 nm. According to the equation, Eg = 1240/λ, the values of the band-gap calculated for CdS is 2.25 eV. The absorption edge in the UV-vis spectrum of the CdS nanoparticle mainly depends on the size of the primary particles^22^. Thus, the variation in the absorption onset of the CdS nanocrystals indicates the change in size. When the size of the CdS nanocrystals becomes smaller than the exciton radius, a remarkable quantum size effect leads to a size-dependent increase in the band-gap and a blue shift in the absorption onset^23^. In comparison to the CdS without L-Histidine sample, a little red-shift of the absorption edge for the CdS with L-Histidine sample can be seen. The shift of the absorption edge toward longer wavelengths indicates a little decrease in the bandgap energy of the CdS. This also implies that CdS with L-Histidine has a smaller band gap, and thus it could efficiently separate the photo-generated electron-hole pairs and improve the visible-light photocatalytic H_2_-production activity.

The specific surface area and porosity of the samples CdS prepared without L-Histidine and with L-Histidine were studied by nitrogen sorption measurements. The BET specific surface area of the CdS prepared with L-Histidine is about 3.32 m^2^g^–1^, which is 1.7 times larger than that of pure CdS. Generally, the photocatalyst with a higher specific surface area is indispensable in the enhancement of photocatalytic performance, due to more surface active sites and photocatalytic reaction centers^24^. Figure 6b presents nitrogen adsorption–desorption isotherms of the samples. According to Brunauer-Deming-Deming-Teller classification, the majority of physisorption isotherms can be grouped into six types^25^. Typically, pure CdS has an isotherm of type II, indicating the presence of large macropores, while CdS prepared with L-Histidine has isotherms of type IV and the shape of the hysteresis loops is of type H3, suggesting the presence of mesopores. In the following discussion section, we will show that the enhanced photoactivity is primarily attributed to the improved lifetime and transfer of photogenerated charge carriers. It has been reported that for photocatalysts with small particles^26^ or nanoporous structures^27^, the distance that the photogenerated electrons and holes have to migrate to the reaction sites on the surface shortens, which benefits the suppression of the electron/hole recombination in the bulk.

## Discussion

**Figure 7:**
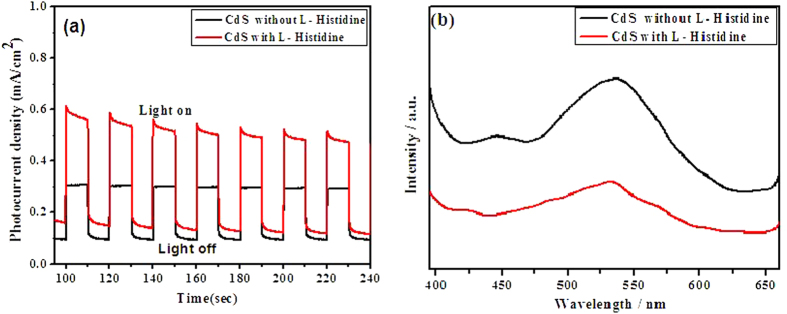
The transient photocurrent of CdS prepared without L-Histidine and with L-Histidine under visible light (a) PL spectra of the as-prepared photocatalysts (b).

The photoelectrochemical performance of semiconductors mainly depends on the generation of photoinduced electron, separation of electron–hole pairs and efficiency of charge carrier transfer^28^. The photocurrent of the samples was measured under the visible-light irradiation as shown in Figure 7a. When the visible light is regularly switched on and off every ten seconds, a series of almost identical electric signals could be obtained. The CdS with L-Histidine has a remarkable increase in current density. The initial current density of CdS without L-Histidine is 0.2 μA/cm^2^, whereas the value for the CdS with L-Histidine is 2 times higher than that of CdS without L-Histidine. This can be attributed to the fact that the photoinduced charge carriers of CdS with L-Histidine are separated more efficiently than in CdS without L-Histidine^29^,^30^.

Photoluminescence (PL) analysis is also employed to investigate the migration, transfer and separation efficiencies of photo-generated electrons and holes in semiconductors, since PL emission of semiconductor mainly arises from the charge carrier recombination^31^,^32^,^33^,^34^,^35^. Figure 7b shows the PL spectra of the CdS photocatalysts without L-Histidine and with L-Histidine. As shown in Figure 7b, the CdS with L-Histidine gives a similar band-to-band fluorescence emission characteristic as CdS without L-Histidine. The two bands for the samples are very typical for CdS nanomaterials. The band centered at around 495 nm is the band-gap emission of CdS nanoparticles, and the broad PL peak between 538–545 nm is commonly attributed to the recombination of the charge carriers in the surface states. Under the same intensity of excitation irradiation, the lower emission intensity implies the better separation of photo-generated electrons and holes pairs of semiconductor photocatalysts. As shown in Figure 7b, much weakened emission intensity of CdS with L-Histidine, compared to pure CdS under the same intensity of excitation irradiation, indicates a greatly decreased recombination probability of photoexcited charge carriers in CdS with L-Histidine.

**Figure 8:**
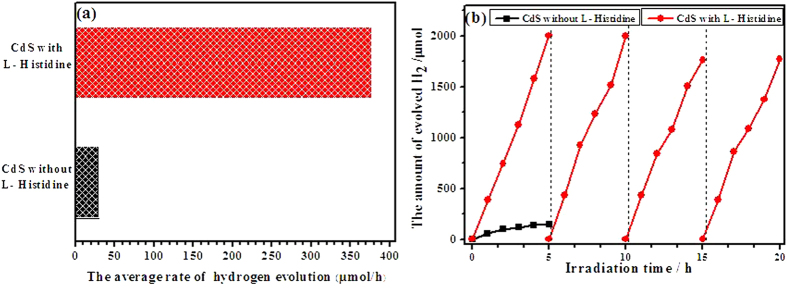
The rate of H_2_ evolution on the samples CdS prepared without L-Histidine and with L-Histidine under visible light (a) Photoevolution of H_2_ on the photocatalysts under visible light irradiation (b).

Figure 8a shows the photocatalytic activities of the samples for hydrogen generation under visible-light irradiation in an aqueous solution containing 0.5 M Na_2_S and 0.5 M Na_2_SO_3_ as the sacrificial reagents. Expectedly, for CdS with L-Histidine, the photocatalytic H_2_-production rate is markedly enhanced to 376.7 μmol/h, nearly 13 times of pure CdS (29.2 μmol/h). Because CdS with L-Histidine has a higher specific surface area and possesses more surface active sites and photocatalytic reaction centers, it exhibits a better photocatalytic performance to produce H_2_. Meanwhile, the photoelectrochemical and PL spectra validate that photo-generated electron-hole pairs of CdS with L-Histidine can be separated more efficiently than pure CdS. These behaviors are beneficial for photocatalytic performance to produce H_2_ and are the mainly reason for higher photocatalytic activity of flower-like CdS, while the detailed mechanism study still needs further investigations. Additionally, platinum (Pt), as an excellent co-catalyst, is loaded onto the CdS samples via a photodeposition method. The experimental result (seeing the Figure S2) shows that the photocatalytic H_2_-production rate of dendritic CdS decorated with 0.3% Pt is 53.1 μmol/h, while the flower-like CdS is 731.1 μmol/h, having a high photocatalytic activity for H_2_-production. In order to check the reproducibility of photocatalytic material and remove the dissolved gases, the reactor was evacuated and the photocatalytic experiment was repeated in every 5 h of reaction (Fig. 8b). The activity of CdS with L-Histidine is found to be almost the same in four repeated runs. The initial H_2_-production rate reaches 2001 μmol/h. In the next run, the rate of hydrogen evolution mildly declines. However, the hydrogen evolution rate still remains 1766 μmol/h in the fourth reaction run, but pure CdS almost does not produce H_2_ after 3 hours, suggesting that the CdS with L-Histidine is more stable than CdS without L-Histidine in the hydrogen generation, which is related to the structure of L-Histidine, the lone pair electrons of amidogen and hydroxy(-NH, -NH_2_ and -OH) in L-Histidine can draw the photogenerated holes of CdS, so the introduction of L-Histidine into the system can remove holes efficiently and avoid the photocorrosion problem of CdS. The slight decrease in the rate of hydrogen evolution might be related to the deactivation of the photocatalyst or attributed to the consumption of the sacrificial reagents in the solution^36^.

In summary, we have successfully synthesized the novel three-dimensional (3D) flower-like CdS nanoarchitectures via a facile template-free hydrothermal process using L-Histidine as a chelating agent. The pure CdS and flower-like CdS samples present regular hexagonal and cubic phases structure. Based on the SEM studies, we propose the growth mechanism of flower-like CdS nanoarchitectures. The imidazole ring of L-Histidine captures the Cd ions from the solution, and prevents the growth of the CdS nanoparticles. The as-synthesized flower-like CdS sample slightly extends the photoresponse to visible light region and exhibits an excellent photoelectrochemical performance. The photocurrent of flower-like CdS is 2 times of pure CdS. Enhancement in photocurrent displayed by flower-like CdS is attributed to the reduction in the electron-hole pairs recombination process. The above factors are assumed to be the main origins that lead to the enhanced photocatalytic activity of the flower-like CdS sample in hydrogen generation under visible light illumination. The flower-like CdS photocatalytic H_2_-evolution rate is 376.7 μmol/h under visible light irradiation, which is 13 times of that of pure CdS.

## Methods

### Materials

Cadium nitrate tetrahydrate (Cd(NO_3_)_2_.4H_2_O, 99.0%), Thiourea (H_2_NCSNH_2_, 99.0%), L-Histidine (C_6_H_9_N_3_O_2_), Dehydrated Alcohol (CH_3_CH_2_OH, 99.7%), Sodium sulfate anhydrous (Na_2_SO_4_, 99.0%), Sodium sulfide (Na_2_S.9H_2_O, 98.0%), Sodium sulfite anhydrous (Na_2_SO_3_, 97.0%) were purchased from Sinopharm chemical Reagent Co., Ltd. All the chemicals were used as received without further purification. Deionized (DI) water was used in all experiments.

### Synthesis of pure CdS

In a typical procedure, 1 mmol of Cd(NO_3_)_2_•4H_2_O, and 3 mmol of thiourea were dispersed in 50 mL distilled water. Next, the homogeneous solution was transferred into a 100 mL Teflon-lined autoclave and held at 180 ^o^C for 4 h after vigorous stirring and sonication. After that, the precipitates were collected by centrifugation and washed with distilled water and ethanol several times. The final product was dried in an oven at 80 ^o^C for 12 h.

### Synthesis of flower-like CdS

In a typical procedure, 1 mmol of Cd(NO_3_)_2_•4H_2_O, 3 mmol of thiourea, and 0.3 mmol of L-Histidine were dispersed in 50 mL distilled water. Next, the homogeneous solution was transferred into a 100 mL Teflon-lined autoclave and held at 180 ^o^C for 4 h after vigorous stirring and sonication. After that, the precipitates were collected by centrifugation and washed with distilled water and ethanol several times. The final product was dried in an oven at 80 ^o^C for 12 h.

### Characterizations

X-ray diffraction patterns (XRD) of the samples prepared were recorded on a Rigaku X-ray diffractometer D/MAX-2200/PC equipped with Cu K_α_ radiation(40 kV, 20 mA). Scanning electron microscopy (SEM) images were obtained using Carl Zeiss Ultra Plus. Transmission electron microscope (TEM) study was carried out on a TECNAI TF20 instrument. UV–vis absorption spectra were measured using Shimadzu UV-3100 spectrophotometer. Nitrogen adsorption-desorption isotherms and the Brunauer-Emmett-Teller (BET) surface areas were collected at 77 K on a TriStar II 3020 system. Steady and time-resolved fluorescence emission spectra were recorded at room temperature with a fluorescence spectrophotometer (PE, LS-55).

### Electrochemical measurements

The photocurrent of the photocatalysts were measured by electrochemical works station (CHI 650E Chenhua, Shanghai, China), using a 300 W Xe lamp(Aulight, CEL-HXF300) which was equipped with an optical filter (0.1 M NaNO_2_ aqueous solution) to cut off the light in the UV region. The fabrication of the working electrodes refers to the reported literature^37^, and the samples were dispersed ultrasonically in absolute ethanol with a concentration of 1.0 g/L. Ten drops of the suspension were put onto a piece of transparent FTO conducting glass (1 × 1 cm^2^) and then dried slowly in room temperature until a layer of film formed on the surface. The electrolyte solution used for all measurements was 0.5 M Na_2_SO_4_. Pt electrode and Ag/AgCl electrode were used as the counter and reference electrodes, respectively. The photocurrent responses of the working electrodes were recorded by sudden light on and off under visible light illumination at the bias voltage of 0.5 V.

### Measurement of photocatalytic activity

The photocatalytic reactions were carried out in a Pyrex reaction cell. 0.15 g powder was dispersed in 100 mL aqueous solution containing 0.5 M Na_2_S and 0.5 M Na_2_SO_3_ as the sacrificial reagents. The suspension was then thoroughly degassed and irradiated by a 300 W Xe lamp(Aulight, CEL-HXF300) which was equipped with an optical filter (0.1 M NaNO_2_ aqueous solution) to cut off the light in the UV region. The amounts of H_2_ evolution were measured by using a gas chromatography (QC-9101, 5 Å-coloum) with thermal conductivity detector(TCD) and Ar as carrier gas.

## Additional Information

**How to cite this article**: Wang, Q. *et al.* Highly Efficient Photocatalytic Hydrogen Production of Flower-like Cadmium Sulfide Decorated by Histidine. *Sci. Rep.* **5**, 13593; doi: 10.1038/srep13593 (2015).

## Supplementary Material

Supplementary Information
