# Supplementary material for: Highly Efficient Photocatalytic Hydrogen Production of Flower-like Cadmium Sulfide Decorated by Histidine
Source: Sci Rep. 2015 Sep 4;5:13593. doi: 10.1038/srep13593 (PMC4559807; doi:10.1038/srep13593)
Supplement: Supplementary Information [file srep13593-s1.doc]

**Supplementary information**

**Highly Efficient Photocatalytic Hydrogen Production of Flower-like Cadmium Sulfide Decorated by Histidine**

Qizhao Wang^a^, ^c^*, Juhong Lian^a^, Jiajia Li^a^, Rongfang Wang^a^, Haohao Huang^b^, *, Bitao Su^a^, Ziqiang Lei^a^

*^a^**College of Chemistry and Chemical Engineering, Northwest Normal University, Key Laboratory of Eco-Environment-Related Polymer Materials, Ministry of Education of China, Key Laboratory of Gansu Polymer Materials, Lanzhou 730070, China*

*^b^College of Materials Science and Engineering, South China University of Technology, Guangzhou, 510640, China*

*^c^Key Laboratory of Green Catalysis of Higher Education Institutes of Sichuan, College of Chemistry and Pharmaceutical Engineering, Sichuan University of Science and Engineering, Zigong, 643000, China*

***Corresponding auther. Tel: +86 931 7972677; Fax: +86 931 7972677.

E-mail addresses: wangqizhao@163.com; qizhaosjtu@gmail.com(Q. Wang)

scuthhh@hotmail.com; hhhuang@scut.edu.cn (H. Huang)

1. **Measuring the apparent quantum efﬁciency (QE)**

We have tried to measure the quantum efficiency of flower-like CdS, but due to the limited apparatus, we can not get the number of incident photons, so we measured the quantum efficiency of flower-like CdS roughly.

the QE is calculated according to the formula. QE%=(number of evolved H_2_ molecules×2/number of incident photons)×100%
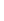
, The number of incident photons is 3.54×10^17^ photons/s , which is the cited value because of alike 300W Xe lamp [1]. The probable quantum efficiency of pure CdS and flower-like CdS is 0.27% and 4.35% at 420 nm, respectively.

1. **Additional figures**


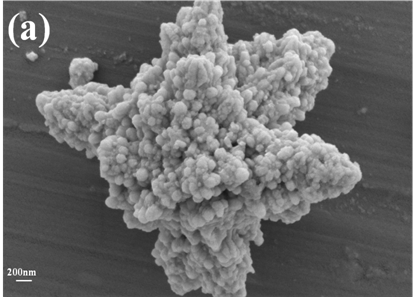

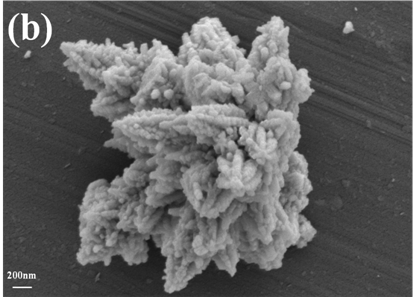


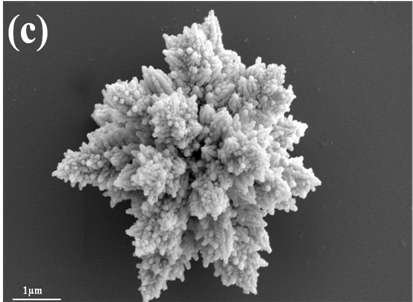

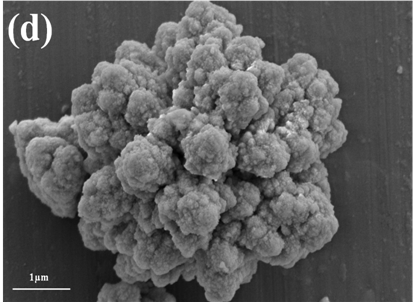


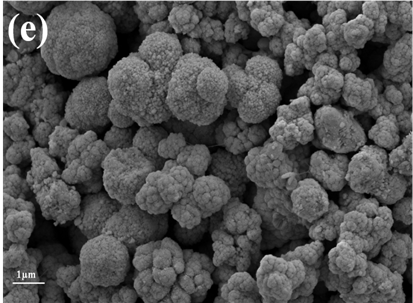


Figure 1 SEM images of CdS prepared with different ratios of Cd precursor to histidine: 10:1 (a); 10:2 (b); 10:3(c); 10:5(d); 10:10(e).

Figure 2 The rate of H_2_ evolution on the samples 0.3% Pt/CdS prepared without L-Histidine and with L-Histidine under visible light.

**Reference:**

1. H. Yan *et al*. Visible-light-driven hydrogen production with extremely high quantum efﬁciency on Pt–PdS/CdS photocatalyst. *Journal of Catalysis* **266**, 165–168 (2009).
